# Supplementary material for: One-year in: COVID-19 research at the international level in CORD-19 data
Source: PLoS One. 2022 May 25;17(5):e0261624. doi: 10.1371/journal.pone.0261624 (PMC9132347; doi:10.1371/journal.pone.0261624)
Supplement: S1 Appendix — (DOCX) [file pone.0261624.s001.docx]

# Appendix A – Network visuals


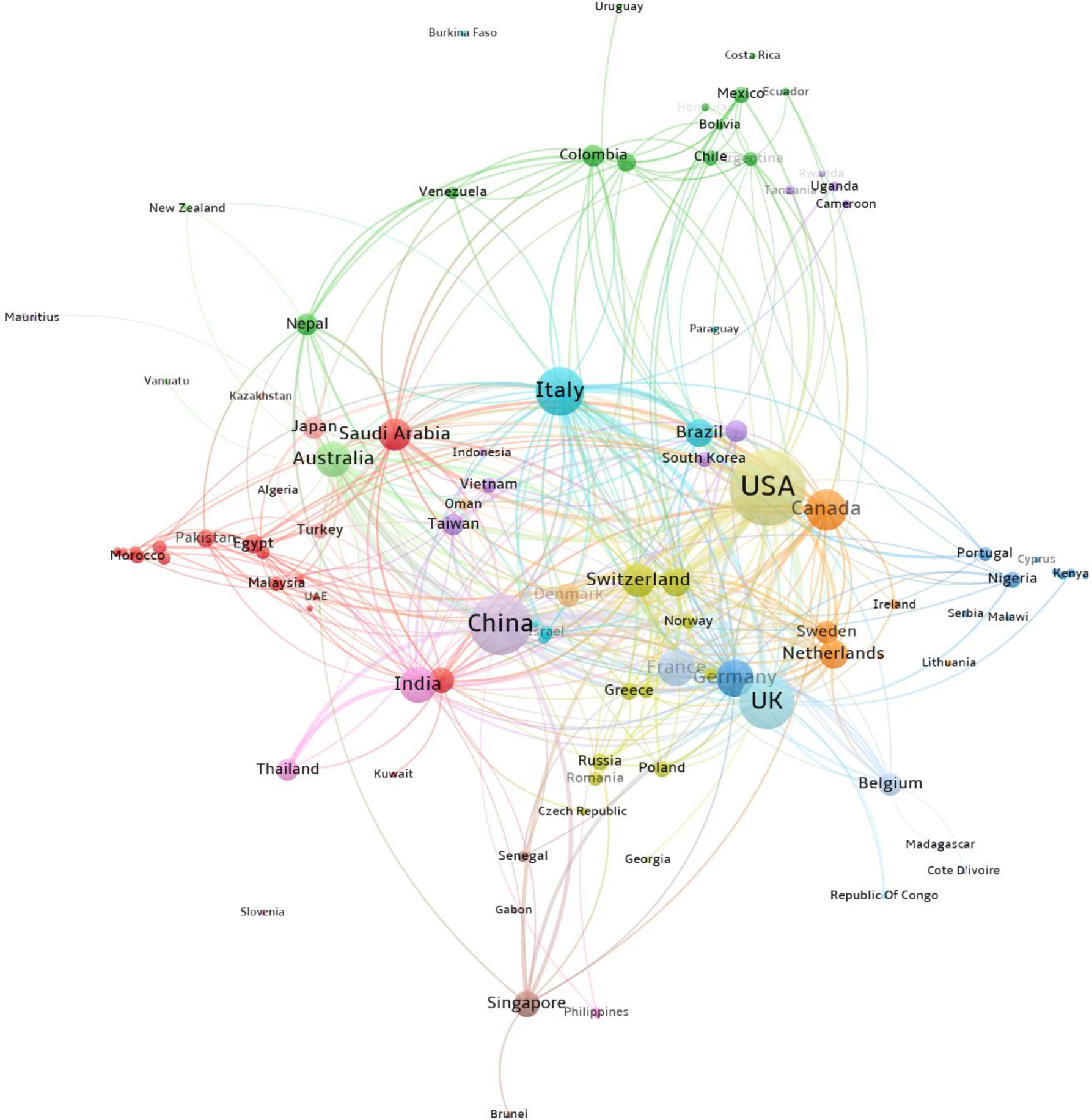


Figure A1. Global Network of international collaborative relationships between January and March 2020.

Interactive version accessible at https://app.vosviewer.com/?json=https://drive.google.com/uc?id=15Tm65Or1n2OYvxOR3nlrhh1fKu_MC1uv


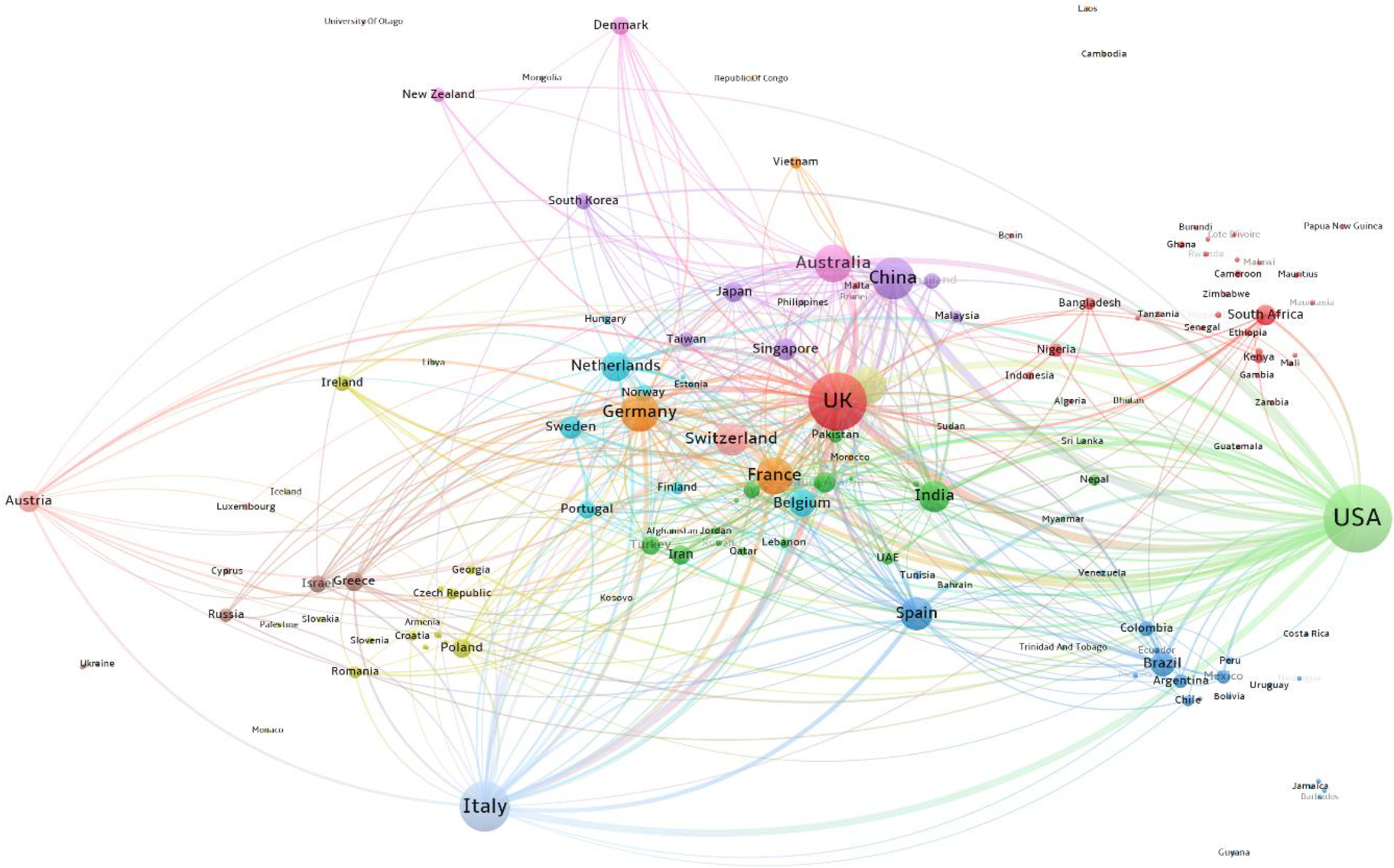


Figure A2. Global Network of international collaborative relationships between April and June 2020.

Interactive version accessible at https://app.vosviewer.com/?json=https://drive.google.com/uc?id=1xF4G-NSwQqP937s9bGlf3CzOIjF9AA0D


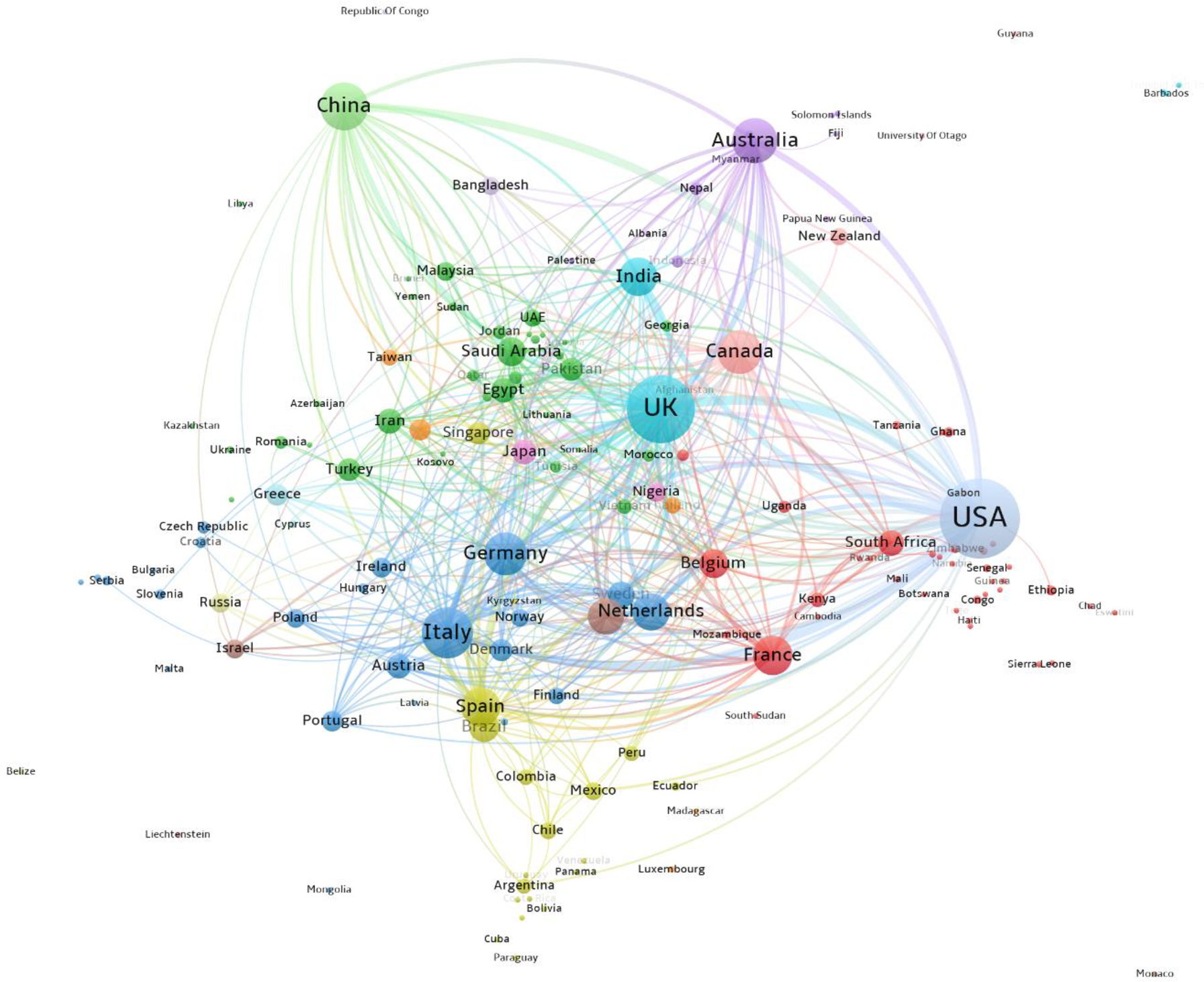


Figure A3. Global Network of international collaborative relationships between July and September 2020.

Interactive version accessible at https://app.vosviewer.com/?json=https://drive.google.com/uc?id=1kk2sQvrWCB1xfFuGepzaDqc6cyyr3MvH


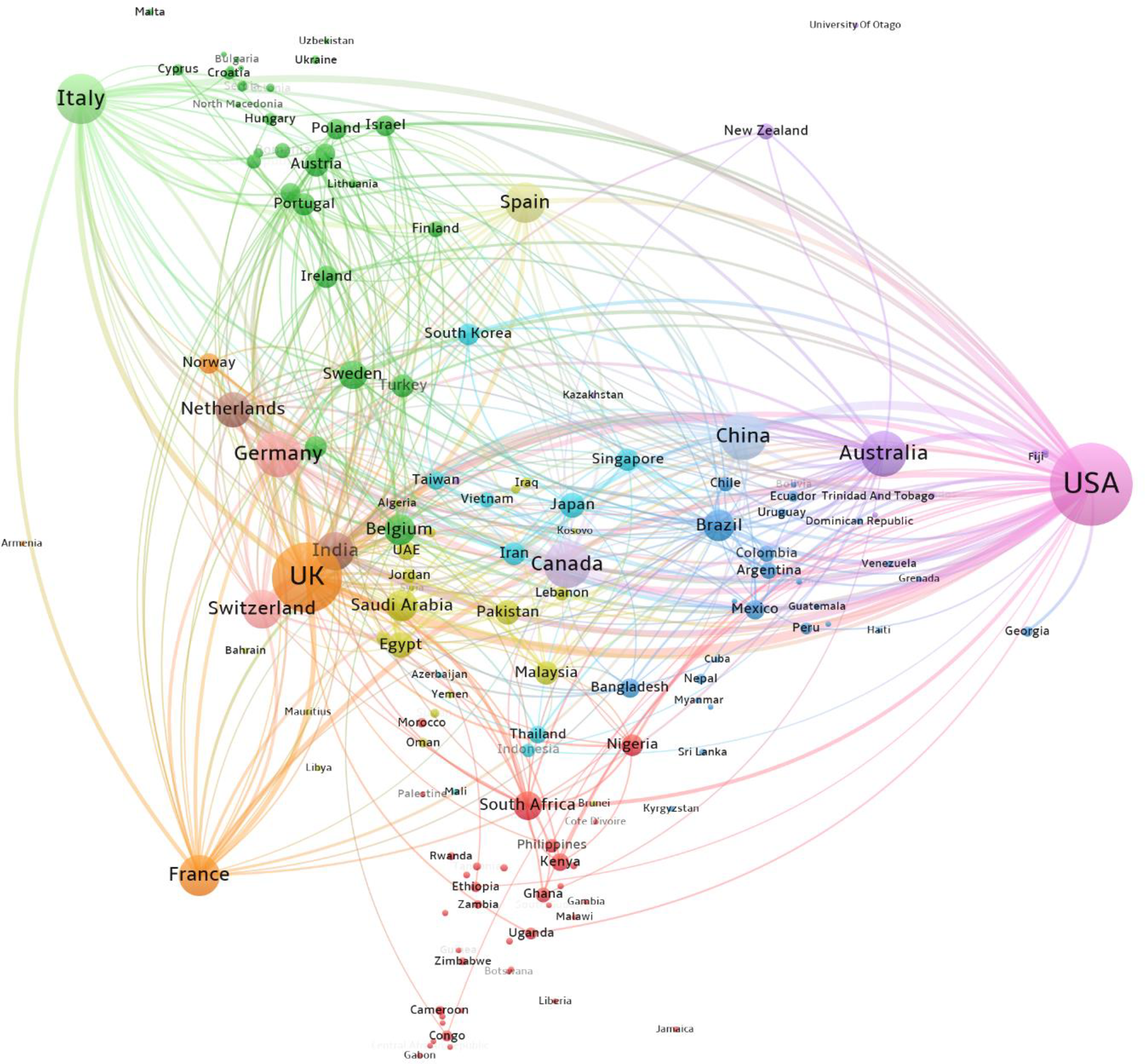


Figure A4. Global Network of international collaborative relationships between October to December 2020.

Interactive version accessible at https://app.vosviewer.com/?json=https://drive.google.com/uc?id=1oD9-RQiHWyghU4SnUAWTiZGDdI_EC886

# Appendix Table 1

Numbers of coronavirus researchers, before and during the COVID-19 pandemic

|  | | Number of publications | Number of distinct authors | Sum of number of authors |
| --- | --- | --- | --- | --- |
| 2015-2019 | All coronavirus articles | 5,530 | 22,264 | 37,202 |
|  | International-collaborated coronavirus articles | 1,807 | 10,036 | 15,033 |
| 2019 | All coronavirus articles | 1,138 | 6,285 | 7,915 |
|  | International-collaborated coronavirus articles | 385 | 2,774 | 3,359 |
| 2020 | All coronavirus articles | 34,767 | 156,295 | 257,677 |
|  | International-collaborated coronavirus articles | 8,580 | 58,970 | 81,640 |

# Source: Web of Science

# Appendix Table 2

Geographical distance across countries in COVID research

|  | (1) | (2) | (3) | (4) | (5) |
| --- | --- | --- | --- | --- | --- |
|  | All country pairs | Excluding China-USA pairs | Pairs without USA | Pairs without China | Pairs with USA or China |
| DV: Salton’s measure |  |  |  |  |  |
| COVID | -0.1112^***^ | -0.1114^***^ | -0.1127^***^ | -0.1125^***^ | -0.1135^***^ |
|  | (0.0075) | (0.0075) | (0.0077) | (0.0076) | (0.0078) |
| Geographic distance | -0.0011^***^ | -0.0011^***^ | -0.0012^***^ | -0.0011^***^ | -0.0011^***^ |
|  | (0.0001) | (0.0001) | (0.0001) | (0.0001) | (0.0001) |
| COVID#Geographic distance | 0.0003^***^ | 0.0003^***^ | 0.0003^***^ | 0.0003^***^ | 0.0003^***^ |
|  | (0.0001) | (0.0001) | (0.0001) | (0.0001) | (0.0001) |
| _cons | 0.2826^***^ | 0.2829^***^ | 0.2856^***^ | 0.2839^***^ | 0.2863^***^ |
|  | (0.0069) | (0.0069) | (0.0071) | (0.0070) | (0.0071) |
| *N* | 4,797 | 4,795 | 4,553 | 4,628 | 4,386 |
| F | 684 | 687 | 669 | 672 | 652 |
| R^2^ | 0.3495 | 0.3505 | 0.3562 | 0.3536 | 0.3593 |

Standard robust errors in parentheses

* p < 0.1, ** p < 0.05, *** p < 0.01

Note: Regressions are run with a model at the country pair level for pre-COVID and COVID-19 collaborative publications. We apply a square root transformation to Salton’s measure and the geographic distance.
