# Supplementary material for: One-year in: COVID-19 research at the international level in CORD-19 data
Source: PLoS One. 2022 May 25;17(5):e0261624. doi: 10.1371/journal.pone.0261624 (PMC9132347; doi:10.1371/journal.pone.0261624)
Supplement: Attachment — Submitted filename: PLOS_R3_12_2_21_response.docx [file pone.0261624.s003.docx]

November 29, 2021

Editors, PLoS One

Submission: PONE-D-21-22845R1, One Year In: COVID-19 Research at the International Level in CORD-19 Data

Dear Editors,

Thank you for this opportunity to revise the referenced article. We have fully responded to the reviewers’ comments. We appreciate the careful attention given by the reviewers to this work. The referee comments have greatly improved the article.

Thank you for requesting the reference to the National Science Foundation data. We have included a reference to the National Science Board indicators report.

*From referee: The discussion of the data and methodology is more clear in the revised paper. I just want to note that it seems to me that the approach taken by the authors, in which Web of Science, Scopus, and Dimensions data were combined, is rather complicated. The use of only one of these three databases instead of all three would probably have been sufficient to carry out all the analyses reported in the paper.*

The reviewer asked why we use three data sets. We apologize if we have not been clear about the data. We have revised the paper to make this clearer. Please note that this team used combined data sets only in the earlier paper, but not in this one. (There, we used preprint and published works, for example.) In the paper currently under review, we considered the use of funding data from the Web of Science because these data were not available from CORD-19. We pulled funding data from the Web of Science and publication data rom CORD-19. We have added text to make these data sets clear.

We sought to correct the figshare link, as requested. We were not able to connect the network data on figshare because the files are too large. The figshare data is the cleaned data from CORD-19.

*Subsection headings don’t seem to have a consistent layout. For instance, ‘Contributions by Author Location’ has a bold formatting, while ‘Co-occurrence network on coronavirus research’ is formatted in italics.*

Thank you for this suggestion. We revised the headings.

*I would like to suggest changing the order of Table 2 and Figure 1. I think it’s more natural to first present Figure 1 and then Table 2.*

Thank you for this suggestion - we changed the ordering.

*“We can assume that the COVID-19 articles were written in 2020, since they are topical—“COVID” was not a keyword in 2019”: The second part of this sentence is unclear. What do you mean by saying that ‘COVID’ was not a keyword in 2019? I am also confused because the search terms listed in the Data and Methodology section include the term ‘coronavirus’, and coronavirus research took place not only in 2020 but also in earlier years.*

We used the term ‘coronavirus’ for all years; COVID is only used for 2020. This is clarified in the text.

*“while the COVID-19 cases also stabilized relative to the earliest months”: This is not correct. The number of cases keeps increasing in Figure 1. Keep in mind that you are using a logarithmic scale. Hence, while the increase may seem small, it is actually a rather large increase.*

We changed the phrasing in the text to: "while the rate of growth in COVID-19 cases declined slightly relative to the earliest months"

*“However, during COVID-19 period, the negative impact of geographic distance on collaboration strength was weakened as demonstrated by positive and significant coefficient of interaction of COVID and geographic distance”: It is not clear which coefficient of interaction you are referring to.*

Thank you for your question. We included an endnote with a link to the table with the coefficients calculated to assess the impact of distance on the likelihood of cooperation.

*“Our visual scan indicates that the additional work, over and above the life sciences and biological inquiries, came in the form of public health and patient-care topics specific to the pandemic, which would not have been part of the pre-COVID research.”: Again it is not clear what you are referring to. Which visual scan do you mean?*

We deleted this sentence since it is not critical to the article.

*Tables 7 and 8: If you want to include betweenness in these tables, you need to explain how it was calculated and how it can be interpreted. In Table 8 betweenness is referred to as ‘weighted betweenness’, while in Table 7 it is just called ‘betweenness’, so it is not clear whether the weights of the links between countries were taken into account in the calculation of betweenness or not. A detailed explanation is necessary, both for the calculation and for the interpretation of betweenness. Interpretation of betweenness is challenging especially in weighted networks. Alternatively, you may consider removing betweenness from the tables.*

We had used ‘betweenness’ measure from Table 7 as it was not central to the argument. Centrality in Table 8 to measure the importance of these selected countries in international collaborations. We explained in the text the reason why we chose this indicator and how we calculated; new text is on Page 12 of the revised draft.

*“showing that influence across the network becomes more diffused as researchers from more countries joined into the research”: It is not clear to me what you mean by influence becoming more diffused.*

We changed the phrasing; "but betweenness centrality drops as researchers from more countries joined into the research"

*In the limitations section, my suggestion is to mention the exclusion of preprints as a limitation. Preprints have played an important role in the rapid dissemination of COVID-19 research, so their exclusion from your work represents a significant limitation.*

We added: “We also exclude preprint publications from the analysis which could present a limitation, given the important role of preprints during the pandemic.”

*“where internationally coauthored articles often account for more than one-third of articles.”: Could you add references to substantiate this statement?*

This is changed to one-quarter and a reference to the National Science Board report has been added.

Thank you again for the careful review and thoughtful comments and suggestions. We appreciate it very much.

Caroline S. Wagner on behalf of myself and coauthors
